# Cytokine storm in aged people with CoV-2: possible role of vitamins as therapy or preventive strategy

**DOI:** 10.1007/s40520-020-01669-y

**Published:** 2020-08-31

**Authors:** Sirio Fiorino, Claudio Gallo, Maddalena Zippi, Sergio Sabbatani, Roberto Manfredi, Renzo Moretti, Elisa Fogacci, Caterina Maggioli, Francesca Travasoni Loffredo, Enrico Giampieri, Ivan Corazza, Christoph Dickmans, Claudio Denitto, Michele Cammarosano, Michele Battilana, Paolo Emilio Orlandi, Francesco Del Forno, Francesco Miceli, Michela Visani, Giorgia Acquaviva, Antonio De Leo, Paolo Leandri, Wandong Hong, Thomas Brand, Giovanni Tallini, Elio Jovine, Roberto Jovine, Dario de Biase

**Affiliations:** 1 UO of Internal Medicine Unit, Hospital of Budrio, Via Benni 44, 40065 Budrio, Bologna, Italy; 2 Physician Specialist in Infectious Diseases, AUSL Bologna, Bologna, Italy; 3 Unit of Gastroenterology and Digestive Endoscopy, Sandro Petrini Hospital, Rome, Italy; 4 Infective Disease Unit, University of Bologna, Bologna, Italy; 5 Experimental, Diagnostic and Specialty Medicine Department, University of Bologna, Bologna, Italy; 6 Unit of Radiology, Maggiore Hospital of Bologna, Bologna, Italy; 7 Internal Medicine Unit, Maggiore Hospital of Bologna, Bologna, Italy; 8 UO Farmacia Centralizzata OM, Farmacia Ospedale Di Budrio, Budrio, Bologna, Italy; 9 Department of Pharmacy and Biotechnology (FABIT), University of Bologna, Bologna, Italy; 10 Department of Medicine (Dipartimento di Medicina Specialistica, Diagnostica e Sperimentale), Molecular Diagnostic Unit, University of Bologna, Azienda USL di Bologna, Bologna, Italy; 11 Department of Gastroenterology and Hepatology, First Affiliated Hospital of Wenzhou Medical University, Wenzhou, Zhejiang The People’s Republic of China; 12 Regenerative Medicine Center Utrecht, University of Utrecht, Utrecht, Netherlands; 13 Surgery Unit, Maggiore Hospital, Bologna, Italy; 14 Physical Medicine and Rehabilitation Unit, Maggiore Hospital, Bologna, Italy

**Keywords:** SARS, COVID-19, CoV-2, Vitamins, Therapeutic strategy

## Abstract

**Background:**

In December 2019, a novel human-infecting coronavirus, SARS-CoV-2, had emerged. The WHO has classified the epidemic as a “public health emergency of international concern”. A dramatic situation has unfolded with thousands of deaths, occurring mainly in the aged and very ill people. Epidemiological studies suggest that immune system function is impaired in elderly individuals and these subjects often present a deficiency in fat-soluble and hydrosoluble vitamins.

**Methods:**

We searched for reviews describing the characteristics of autoimmune diseases and the available therapeutic protocols for their treatment. We set them as a paradigm with the purpose to uncover common pathogenetic mechanisms between these pathological conditions and SARS-CoV-2 infection. Furthermore, we searched for studies describing the possible efficacy of vitamins A, D, E, and C in improving the immune system function.

**Results:**

SARS-CoV-2 infection induces strong immune system dysfunction characterized by the development of an intense proinflammatory response in the host, and the development of a life-threatening condition defined as cytokine release syndrome (CRS). This leads to acute respiratory syndrome (ARDS), mainly in aged people. High mortality and lethality rates have been observed in elderly subjects with CoV-2-related infection.

**Conclusions:**

Vitamins may shift the proinflammatory Th17-mediated immune response arising in autoimmune diseases towards a T-cell regulatory phenotype. This review discusses the possible activity of vitamins A, D, E, and C in restoring normal antiviral immune system function and the potential therapeutic role of these micronutrients as part of a therapeutic strategy against SARS-CoV-2 infection.

## Introduction

In December 2019, a novel human-infecting coronavirus, defined SARS-CoV-2 or 2019-nCoV, had been recognized as a very severe life-threatening health problem in Wuhan, Hubei Province in China. This infectious condition is now known as “coronavirus disease 2019” (abbreviated “COVID-19”); its most frequent manifestation is represented by the development of pneumonias with different forms of severity [1]. However, less common symptoms, such as diarrhea, headache, myalgia or arthralgia, chills, nausea or vomiting, nasal congestion and conjunctival congestion, have been described in infected individuals. From China, the epidemic has spread worldwide, and the number of subjects infected with the virus is progressively growing every day. On January 30, 2020, the International Health Regulations Emergency Committee of the World Health Organization classified the epidemic as a “public health emergency of international concern” [2]. A dramatic situation is progressively emerging in Italy with an increasing number of infected subjects, who suffer from severe forms of interstitial pneumonia and are at a high risk of mortality. Therefore, the rising need for intensive care beds with the purpose to provide an effective treatment of these patients could cause the collapse of the Italian Health System and induce similar consequences very quickly in other countries as well, even in the most developed nations. Unfortunately, neither a vaccine nor a proved specific therapy against this virus is currently available. Therefore, new strategies of treatment are strongly needed to efficaciously combat SARS-CoV-2 and to establish effective antiviral schedules. Similar to a wide range of other viruses, SARS-CoV-2 is also able to interact with the host, influencing the antiviral immune response and determining its pathogenesis. It is well known that proteins produced by a large series of viruses may modify the amounts of cell RNA transcripts (and consequently of the codified cell proteins) directly by binding to specific cell DNA sequences or indirectly by activating cytoplasmatic cell signaling pathways. In particular, HBx, NS3, NS5A, and NS5B have been demonstrated to dysregulate the expression profile of some cellular cytoskeletal genes through these mechanisms [3] and to interact with distinct elements of cytoskeleton (microfilaments, microtubules, intermediate filaments and actin stress fibers) [4–7]. Furthermore, some cell nuclear factors may bind to the promoter regions of several viral genes and influence the rate of viral proteins production and viral replication [8, 9].

## Virion and genome structure of SARS-CoV-2

**Fig. 1:**
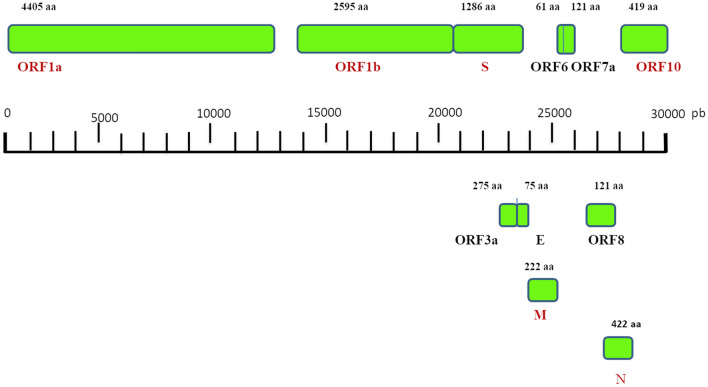
Coronavirus genome with its structural and nonstructural proteins (see in text) is shown. N and S viral proteins, involved in the cytokine release syndrome are shown

SARS-CoV-2, like MERS-CoV and SARS-CoV, consists of an enveloped spherical beta coronavirus with spike proteins projecting from the surface of the viral particles [10]. The envelope is composed of a lipid bilayer originated from the host cell membrane, whereas its genome is characterized by a positive-sense, single-stranded and non-segmented RNA [11]. It is 5′-capped and 3′-polyadenylated RNA, encoding four viral structural proteins: the spike (S) glycoprotein, the matrix (M) protein, the small envelope (E) protein and the nucleocapsid (N) protein. It also includes multiple open reading frames (ORFs), codifying accessory proteins interposed among the structural genes (Fig. 1). The N protein is detectable in the core of the viral particle and it interacts with the CoV RNA, playing a crucial role in the transcription of viral RNA and in the viral nucleocapsid assembly to generate mature virions [12]. The S protein consists of a heavily glycosylated protein generating homotrimer spikes on the surface of the viral particles and modulates the process of viral entry into host cells [13]. Two large overlapping ORFs (ORF1a and ORF1b) encode the coronavirus replicase and occupy about two-thirds of the genome. Furthermore, the viral genome also includes several accessory proteins, such as ORF 3a, 3 b, 4a, 4b, 5a, 5b, 7 and 7a. These have pleiotropic and not well-known effects in the infected hosts. These proteins have been shown to be able to inhibit type I interferon production and type I downstream signaling [14]. These enzymes are directly translated from the genomic RNA, whereas the structural and accessory genes are generated from viral subgenomic RNAs (sgRNAs). These fragments are produced during viral genome transcription/replication [15].

## SARS-CoV-2 immunopathogenesis and role of the different viral proteins of CoVs in the modulation of inflammatory process

On the basis of the immunopathogenic hypothesis by Gu and colleagues [16], and on the assumption that the genome of 2019-nCoV is characterized by about 89% of nucleotide identity with the bat SARS-like-CoVZXC21 and around 82% with that of human SARS-CoV [17], it may be suggested that SARS-CoV-2 immunopathogenesis shares many common aspects with the immunopathogenic events observed during SARS-CoV infection. Therefore, it may be assumed that an inadequate and inappropriate host immune response against this virus may explain the severity of the disease detected in a part of the patients suffering from this pathological condition, which sometimes leads to severe distress acute respiratory syndrome (ARDS) with an unfavorable outcome [18, 19]. Crucial immunopathogenic characteristics of the infectious disease caused by SARS-CoV-2 are represented by the induction of intense immune system dysfunction with the development of a strong proinflammatory host response, following the synthesis and the release of some viral antigens; this event may induce the emergence of a life-threatening condition defined as cytokine release syndrome [20]. The inhibition or the dysregulation of the immune system’s protective responses against SARS-CoV-2, as well as the induction of inefficient activities in different types of immune cells, prevent the effective control of this virus and may induce harmful immune responses, eventually leading to a dismal outcome. Therefore, a deeper knowledge of the immunopathogenesis of this disease is a pressing need and may help in identifying potentially useful therapeutic targets to improve the quality and efficiency of the immune response. On the basis of Gu’s hypothesis [16] and of genome homology between CoV and CoV-2 [17, 18], it may be suggested that the CoV-2 virus also enters the respiratory tract, affecting the epithelial cells of the trachea, bronchi, bronchioles, and lungs. However, the colonization of resident, infiltrating, and circulating immune cells represents the key event in the pathogenesis of this infectious disease. This pathogen is carried by the infected circulating immune cells and reaches the nasal associated lymphoid tissue (NALT), the bronchus-associated lymphoid tissue (BALT) and the mucosa-associated lymphoid tissue (MALT), spreading to the lymphoid tissue of other organs as well as to the mucosa of the intestine, the epithelium of the renal distal tubules and the neurons of the brain. Overall, these events lead to a serious impairment of immune system activities. The severity of the immune cell damage, more than the extent of the lesions detectable in the lungs, suggests that the patient's immune status and his lymphocyte count probably represent the main predictors of his clinical evolution. Viral load also may exert a crucial impact on the strength and efficacy of the patient’s immune response.

## Immune response in aged people and in patients with chronic diseases

**Fig. 2:**
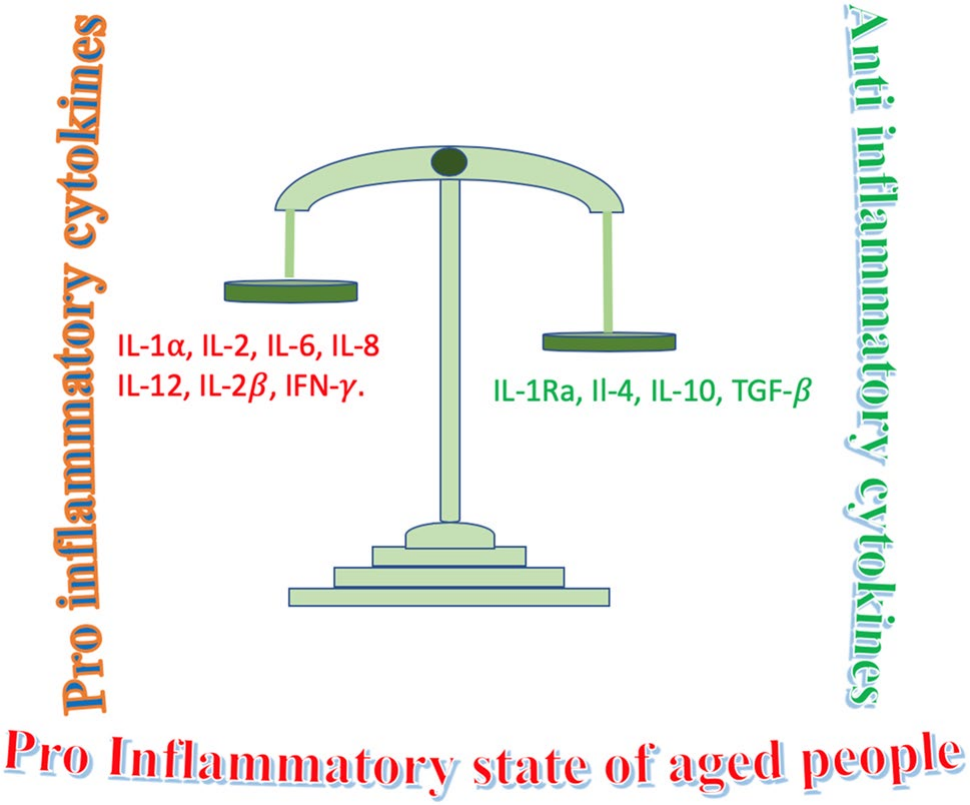
Old individuals present some qualitative/quantitative dysfunctions and defects in their immune response with the establishment of a persistent low-grade inflammatory state. A pro-inflammatory cytokine pattern is generally present in aged people. It is characterized by the emergence of an imbalance in the cytokine pattern with the production of proinflammatory cytokines (IL-1α, IL-2, IL-6, IL-8, IL-12, IFN-γ), which become prevalent in comparison with anti-inflammatory ones (IL-1 Ra, IL-4, IL-10, TGF-β). The strength of the immune response decreases with age, with a modification in the composition and activity of lymphocytes in secondary lymphoid tissues. Furthermore, a large series of deficits emerges in B lymphocytes. They show a decreased ability in responding to viral infections like influenza and in producing antibodies, which show decreased binding activity against their antigens. CD4 positive T-helper cells present dysfunctions in their activation and increased differentiation into Th-17 lineage. Overall, these modifications result in decreased immune activities in the elderly

Patients with chronic diseases, including Alzheimer’s disease, obesity, chronic obstructive pulmonary disease, chronic viral hepatitis, diabetes mellitus and cardiovascular diseases [21–24], as well as old-individuals present some qualitative/quantitative dysfunctions and defects in their immune response with a persistent low-grade inflammatory state, characterized by the establishment of an imbalance in the cytokine pattern with the production of proinflammatory cytokines (IL-1α, IL-2, IL-6, IL-8, IL-12, IFN-γ), which become prevalent in comparison with anti-inflammatory ones (IL-1 Ra, IL-4, IL-10, TGF-β) (Fig. 2) [25]. In brief, the strength of the immune response decreases with age, with a modification in the composition and activity of lymphocytes in secondary lymphoid tissues. Furthermore, a large series of deficits emerge in B lymphocytes. They show decreased ability to respond to viral infections like influenza and produce antibodies with decreased binding activities against their relative antigens. CD4-positive T-helper cells present dysfunctions in their activation and increased differentiation into T helper 17 lineage. CD8-positive T cells show impaired activity, exhibiting oligoclonal expansion and loss of CD28. Increased concentrations of inflammatory cytokines may be produced by stromal elements, dendritic cells, or aging B and T cells. Additional modifications are represented by the enhanced number of memory cells that persistently colonize tissue niches and induce an inflammatory milieu. These events compromise the ability of naive B and T cells to migrate from the bone marrow and thymus towards the peripheral host tissues. Overall, these modifications result in decreased immune activities in the elderly [26–28].

## Serum levels of fat-soluble A, D, E and water-soluble C vitamins in aged people and their role in proper function of normal immune system

Serum levels of different fat-soluble A, D, E, water-soluble C vitamins and other nutrition-related parameters have been associated with frailty in aged people [29, 30]. The prevalence of low vitamin D status represents a global health problem not only in the oldest individuals, but also in all age groups, even in geographical areas with sun exposure all year round [31–33].

It has been reported that there is an association between vitamin D deficiency and a higher risk of intensive care admission [34] and mortality in subjects with more severe forms of pneumonia [35]. Furthermore, vitamin D deficiency has been commonly observed in patients with ARDS, following different causes, including pneumonia, sepsis, pancreatitis, chest trauma or aortic aneurysm repair [36]**.** Some studies have assessed vitamins A, E and C serum levels in different geographical areas, including Italy, both in institutionalized and non-institutionalized elderly people and have detected deficits of variable extent in the amount of fat-soluble and water-soluble vitamins. In particular, about 10–20% and 50% of subjects over 70 years, who were included in the Italian research, had low serum levels of vitamin E and ascorbic acid, respectively [37]. Similar results of vitamin deficiency in elderly people have been described in other reports [38, 39].

The plasma levels of vitamins A, C, E and D were significantly lower in patients with metabolic syndrome compared to healthy subjects [40, 41]. Fat-soluble and water-soluble vitamins are needed for proper functioning and activity of the immune system and their immunoregulatory and immunomodulatory role have been observed and described both in animal and human models in healthy individuals and in patients with different diseases. The deficiency of these micronutrients has been demonstrated to impair normal functioning of the immune system in humans [42–46]. These deficits can be corrected in vivo by providing the supplementation of these vitamins. Several studies have been focused on furthering our understanding of the emergence, development, activity and regulation of dendritic cells (DCs), macrophages, natural killer (NK) cells, T cells, and B cells and of the modulatory effects of vitamins on the specific immune response [47, 48].

## Aim of review

On the basis of the available epidemiological data concerning the current outbreak of the novel SARS-CoV-2 in Italy, the infectious disease caused by this virus represents a very severe health problem for individuals over 60, with aged-people at higher risk of severe forms of disease and of death. About 85% of individuals who died from COVID-19 infection were over the age of 60 (https://www.epicentro.iss.it/, Istituto Superiore Sanità, accessed on 25/3/2020).

Taking advantage of all these epidemiological data, observations, assumptions, and hypotheses, we have compiled this review, with the following aims:

1. to examine the possible aspects of the complex loop which can develop between host and SARS-CoV-2, and the factors and mechanisms involved in this intricate process.
2. the possible immunoregulatory role of fat-soluble and water-soluble vitamins in this life-threatening condition. This strategy may contribute to identify the possible viral targets and to hypothesize a potential therapeutic strategy against this pathogen in a coordinate and consequential way. Unfortunately, it must be considered that this virus has just been isolated recently and that only a few articles describing its structure and genome organization have been published.

## Role of the different viral proteins of CoVs in the modulation of inflammatory process

**Fig. 3:**
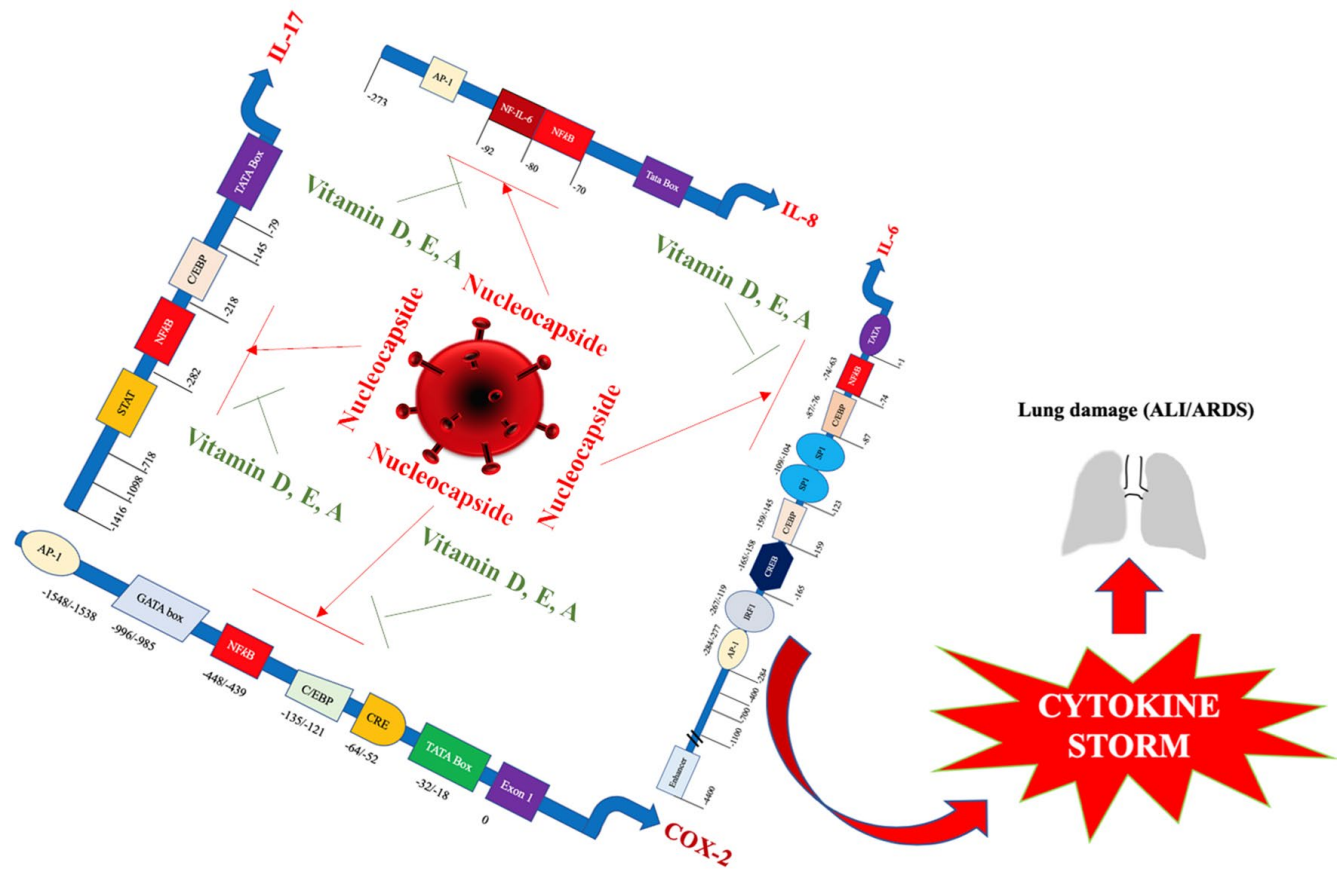
Possible pathogenetic mechanisms involved in cytokine release syndrome in patients with SARS-CoV-2 infection. SARS-coronavirus nucleocapsid and/or spike viral proteins may directly bind to DNA-specific motifs (nuclear factor-kappa B or NF-kB and CCAAT/enhancer binding protein or C/EBP) on the promoters of a wide series of genes encoding proinflammatory cytokines or may interact with specific subdomains in NF-kB protein and activate it. NF-kB is one of the main mediators involved in the generation of the inflammatory process. Therefore, nucleocapsid and/or spike viral proteins may directly or indirectly induce the synthesis of IL-1, IL-6, IL-8, IL-17 and TNF-α during the development of SARS-CoV-2-caused infection. It is conceivable that viral replication rate in infected cells progressively increases during SARS-CoV-2 infection. This event may be associated with the release of elevated amounts of N and S proteins. The high load of these antigens binding to the promoters of the proinflammatory cytokines and enzymes may induce a hyper-activation in the transduction and translation of these genes. Therefore, elevated amounts of proinflammatory cytokines are produced and released. The massive secretion of these mediators is associated with the emergence of the cytokine release syndrome. Aged people suffering from chronic diseases and from immune system function impairment are at high risk of a severe pathological disease. The overall effect of these events is the induction of a proinflammatory pattern leading to a self-maintaining loop with the possible progressive worsening of clinical conditions. Vitamins A, D and E may decrease the binding of NF-κB to their DNA-specific sequences and reduce pro-inflammatory cytokine synthesis, preventing or attenuating the establishment of the cytokine release syndrome

It has been reported that some viral proteins of SARS-CoV are able to modulate the inflammatory process directly or indirectly, by influencing the expression of a large series of cytokines. According to our hypothesis, it is conceivable that SARS-CoV-2 may have common pathogenetic mechanisms with CoV. A lot of host and viral components have been reported to affect this very complex process (Fig. 3). In particular, the induction of the inflammatory response in the patients suffering from SARS-CoV-2 seems to be characterized by a strength, an extent and a duration of cytokine release of different degrees and with different outcomes [49, 50]. According to our knowledge, it is generally thought that about 5–7 days after the infection, SARS-CoV-2 is able to cause the activation of a robust immune response in affected individuals with the production and release of a wide spectrum of proinflammatory cytokines and chemokines [2], leading to the event called “cytokine storm” and to the clinical condition known as “cytokine release syndrome” [51]. According to available epidemiological data in the current situation as well as to Gu’s hypothesis, these events seem to emerge in about 15–20% of infected subjects and have a critical impact on the degree of tissue damage, resulting in the stimulation of its remodeling. Substantial alterations in the anatomy of organs and tissues may emerge and induce considerable modifications in their activity [16, 18]. Overall, in SARS-CoV, and probably in SARS-CoV-2, the patient’s expression of proinflammatory cytokines, including IL-1, IL-2, IL-6, IL-8 and IL-17, and chemokines such as CXCL10 and CCL2 is increased both in peripheral blood and lungs of individuals infected with CoV-SARS, which is associated with disease severity [52]. Several signal transduction cascades are activated during SARS-CoV and SARS-CoV-2, which induce the synthesis and release of proinflammatory cytokines. Among the mediators involved in this process, AP-1 (activating protein), NF-κB (nuclear factor kB) and NF-AT (nuclear factor of activated T cells) are the most studied and known [53]. Furthermore, several NF-AT binding sites have been identified on TNF-α promoter. Overall, these components of inflammatory cascades contribute to amplify the pro-inflammatory response detectable in patients with SARS-CoV infection. All these events have a crucial impact on the outcome of the infection and may explain the differences in the clinical severity of lung damage. Some viral proteins have been proved to stimulate this type of robust pro-inflammatory responses during SARS-CoV infection.

SARS-CoV N protein is able to (Fig. 3):

1. *Activate COX-2 gene expression via the direct interaction with regulatory binding sites* (nuclear factor-kappa B or NF-kB and CCAAT/enhancer binding protein or C/EBP) detectable in the promoter of this gene [54]. The transcription of COX-2 gene, in response to several cellular biochemical mediators such as cytokines, produces an enzyme isoform involved in the active production and release of prostaglandins. This event leads to the up-regulation of the inflammatory process, through the activation of multiple COX-2 signaling cascades and the modification of their function [55]. The induction of COX-2 and release of Prostaglandin E-2 (PGE-2) represent critical events for efficient viral replication in infected hosts. The inhibition of COX-2 decreases viral progeny levels in virus-expressing cultured cell models [56]. In addition, PGE-2 exerts an immune-suppressive effect, modulating both the innate and adaptive components of the immune system [57]. Furthermore, PGE2 exerts a complex regulatory activity on IL-8 gene expression. This function correlates with the concentration of this chemokine as well as with the cell specificity. IL-8 acts as a strong chemoattractant for neutrophils into local inflammatory microenvironment. A large spectrum of cells produces IL-8 in response to different stimuli and contribute to influence the extent of the inflammatory response [58, 59].
2. *Activate IL-1 gene transcription* Interleukin (IL)-1 is a cytokine with pro-inflammatory properties. It is composed of two polypeptides: IL-1a and IL-1b. Its activity is controlled by a natural competitive inhibitor, IL-1 receptor antagonist (IL-1RN) [60]. According to available data, no NF-kappa B binding within cellular IL-1 alpha promoter has been detected, whereas several AP-1-binding sites for Jun and Fos proteins have been isolated. The development of the inflammatory process is characterized by an increased binding of AP-1 proteins to the specific sequences on the DNA, resulting in IL-1 alfa production with consequent pro-inflammatory effects [61].
  A recent study has evaluated the induction of pro-inflammatory cytokines and lung inflammation during SARS-CoV-2 infection. The binding of COVID-19 to the Toll-like receptor (TLR) of immune cells causes the release of pro-IL-1β. This element is cleaved by caspase-1 and induces inflammasome activation and release of active mature IL-1β. This element mediates the development of lung inflammation, fever and fibrosis [62].
3. *Activate IL-6, IL-8 and TNF-α gene transcription* (Fig. 3) Elevated serum levels of IL-6, IL-8 and TNF-α have also been detected in individuals suffering from SARS-CoV infection during the acute stage (cytokine storm) of the disease in association with lung lesions [63]. It has been reported that SARS-coronavirus nucleocapsid and/or spike viral proteins may directly bind to specific DNA motifs on the promoters of a wide series of genes encoding proinflammatory cytokines or may interact with specific subdomains in NF-kB protein and activate it. NF-kB is one of the main mediators involved in the generation of the inflammatory process. Therefore, nucleocapsid and/or spike viral proteins may directly or indirectly induce the synthesis of IL-1, IL-6, IL-8, IL-17 and TNF-α during the development of SARS-CoV-2-caused infection [49]. The overall effect of these events is the induction of a proinflammatory pattern leading to a self-maintaining loop with the possible progressive worsening of clinical conditions [49, 50]. It may be hypothesized that the viral antigen load may have an important impact on the amounts of released interleukins and, consequently, on the extent of the inflammation.

Moreover, ORF 3a, 3b, 4a, 4b, 5a, 5b, 7 and 7a, as well as M, N and S (Fig. 3), have been shown to be able to inhibit both type I interferon production and its downstream signaling and to suppress the antiviral response of the host [52, 64].

## Clinical signs, symptoms and anatomopathological features in patients with SARS-CoV-2 infection

Up to now, the specific factors and pathogenic mechanisms involved in SARS-CoV-2-mediated infection and the transmission route in human hosts are not well understood. According to the available studies, SARS-CoV and SARS-CoV-2 are considered respiratory viruses, associated with elevated morbidity and mortality [65]. The respiratory tract is the main site of infection and the clinical features during the disease caused by SARS-CoV-2 range from an acute respiratory illness with fever, cough and shortness of breath, to more severe forms, such as acute lung injury and in some cases to acute respiratory distress syndrome with a fatal outcome, to septic shock [19, 66–68]. Furthermore, SARS-CoV-2 might also infect the cells of the intestinal mucosa, the epithelium of the renal distal tubules and the neurons of the brain and macrophages in different organs, as suggested by Gu regarding subjects with SARS-CoV. The presence of symptoms such as diarrhea, hematuria, headache and paresthesia are often observed in patients with SARS-CoV-2 and seem to confirm these two viruses share common pathogenetic mechanisms [16]. Autoptic lung samples from subjects who died because of SARS are characterized by some tissue alterations. The most frequent changes include extensive cell infiltrates involving the interstitium and alveoli, with alveolar damage (DAD) and hemorrhage/edema, hyaline membrane development, fibrin exudation, epithelial necrosis with thickening of alveolar septa in the earlier stages of the disease and the emergence of fibrosis in septa and alveoli in the later ones. In particular, DAD is a critical and very important histological feature observed in the lungs from patients who died because of a SARS-CoV-induced infection [16]. Viral genome and antigens have been detected in the epithelial cells of the upper airway and alveoli as well as in vascular endothelial cells, neutrophils, macrophages, monocytes and lymphocytes found in samples obtained from individuals and from animal models [16, 69].

## The current knowledge on the possible activity of the liposoluble and hydrosoluble vitamins alone or in association with other drugs against viral infection

Our current knowledge concerning the immunopathogenesis of SARS-CoV- and SARS-CoV-2-mediated disease suggests that both the type and the quality of the immune response against these pathogens represent crucial factors during the course of this disease and may have a strong impact on the final outcome of the affected subjects [62]. Therefore, the re-modulation and the regulation of the inappropriate and exaggerated pro-inflammatory response observed during SARS-CoV-2 infection may be a key point in the strategy to counteract this virus and to prevent its life-threatening effects. All these considerations may contribute to explaining the differences in clinical course and severity of illness in patients with COVID-19 infection and may offer a conceptual basis for the development of novel therapeutic and/or preventive approaches. As previously reported, in the last years a large series of epidemiological studies have underlined that some fat-soluble and water-soluble vitamins (A, D, E and C) are essential elements for normal immune system function [43–46, 70, 71] and for individuals suffering from a wide spectrum of diseases, including chronic viral infections. It has been suggested that the deficiency of these micronutrients may impair the host’s antiviral defenses and favor the persistence of several viruses-mediated infections [21]. Some studies have been carried out in patients with some chronic viral infections, like HBV, HCV, and HIV. These trials have shown the following in chronically infected patients: (a) serum concentrations of vitamins A, E, D and C are decreased [72, 73], (b) vitamins A, D, E and C deficiency is associated with higher levels of viral replication as well as with higher titers of pro-inflammatory cytokines, like IL-6 and TNF-α, or with illness severity in some studies carried out in different virus-associated diseases [72–79], (c) vitamins may suppress or reduce viral replication or load in different virus infections both in adults and in children [80–84].

## The possible activity of liposoluble and hydrosoluble vitamins in the direct and/or indirect modulation of host immune response by vitamins during SARS-CoV-2 infection

Some of the results obtained with the use of vitamins, alone or in association with different drugs at pharmacological dosages, to counteract both DNA and RNA viruses seem to be encouraging and may provide the rationale for the inclusion of these micronutrients in the multi-therapeutic schedules for the treatment of SARS-CoV-2. In the next parts of this paper, we will examine some of the mechanisms involved in CoV-2-mediated pathogenesis and we will discuss the potential antiviral activities and the possible viral targets of A, D, E and C vitamins.

These micronutrients have been reported to modulate and regulate:

1. *the host-inflammatory status in several chronic diseases* [85], including viral infection. It has been shown that vitamin D and vitamin E, the latter displaying this function mainly in its succinate form, are able to quench reactive oxygen species (ROS) and prevent the activation of a wide series of genes as well as the modification of cytoplasmic enzymatic pathways. Overall, all these components are involved in the induction and control of the inflammatory cascade [86, 87]. Furthermore, vitamin C is able to attenuate ROS-mediated injury in some critical cell micro-organelles, like mitochondria, and to confer protection against oxidative-mediated damage [88]. These mediators are released in the tissue microenvironment, where the inflammatory process originates following the influx of a wide spectrum of different immune cells. In the initial phase of the inflammation, these reactive oxygen species exert a protective role, counteracting the invading pathogens [89]. However, the continuative production of these mediators is associated with the establishment of a persistent self-maintaining pro-oxidative status with the progressive impairment of a lot of cell functions of micro-organelles, including mitochondria and endoplasmic reticulum. These events lead to general tissue damage at a microscopic level and are associated with clinical effects of different severity [90]. Overall, injury involving cell micro-organelles and microenvironment is induced and mediated by the synthesis or by the activation of elements associated with the inflammatory cascade, including NF-kB, AP-1 and PGE2. It has been reported that nucleocapsid and spike proteins of SARS-CoV are able to directly up-regulate the promotor of IL-6, IL-8, IL-12, TNF-α, COX-2 and probably IL-17 genes and indirectly up-regulate those of IL-1 α and β via NF-κB and AP-1 pathways (Figs. 3, 4) [49, 50]. Studies in vitro have shown that 1α,25-dihydroxyvitamin D3 is able to down-regulate DNA binding of nuclear factor-kappa B (NF-κB) to promoters of IL-6, IL-8, IL-12 and COX-2, leading to moderate transcriptional repression with decreased synthesis of all these interleukins and of COX-2 [91–93]. NF-κB motifs have been observed even in IL-1 α and β [94] and IL-17 [95], and it is conceivable that 1α,25-dihydroxyvitamin D3 may also down-regulate the synthesis of these interleukins, although no studies concerning this topic are available. It has been shown that vitamin E is also able to inhibit NF-κB binding activity [96, 97]. Therefore, these micronutrients may down-regulate the synthesis of IL-6, IL-8, IL-12, and COX-2. A significant anti-inflammatory effect of all-trans-retinoic acid (ATRA) on proinflammatory cytokine and chemokine production in adipocyte and adipose tissue model has been observed in models of human and mouse adipocytes, via inhibition of NF-κB [98]. Overall, the use of vitamins A, D, E and C might contribute to decreasing excessive inflammation, which is observed during the development of severe forms of SARS-CoV-2 infection, and to attenuating the cytokine release syndrome. It is not known whether the supplementation of these vitamins at pharmacological dosages in CoV-2 patients, alone or in combination with different associations might induce the significant inhibitory effects on the synthesis of the above-mentioned interleukins. Well-designed trials with the purpose to clarify this hypothesis are needed.

**Fig. 4:**
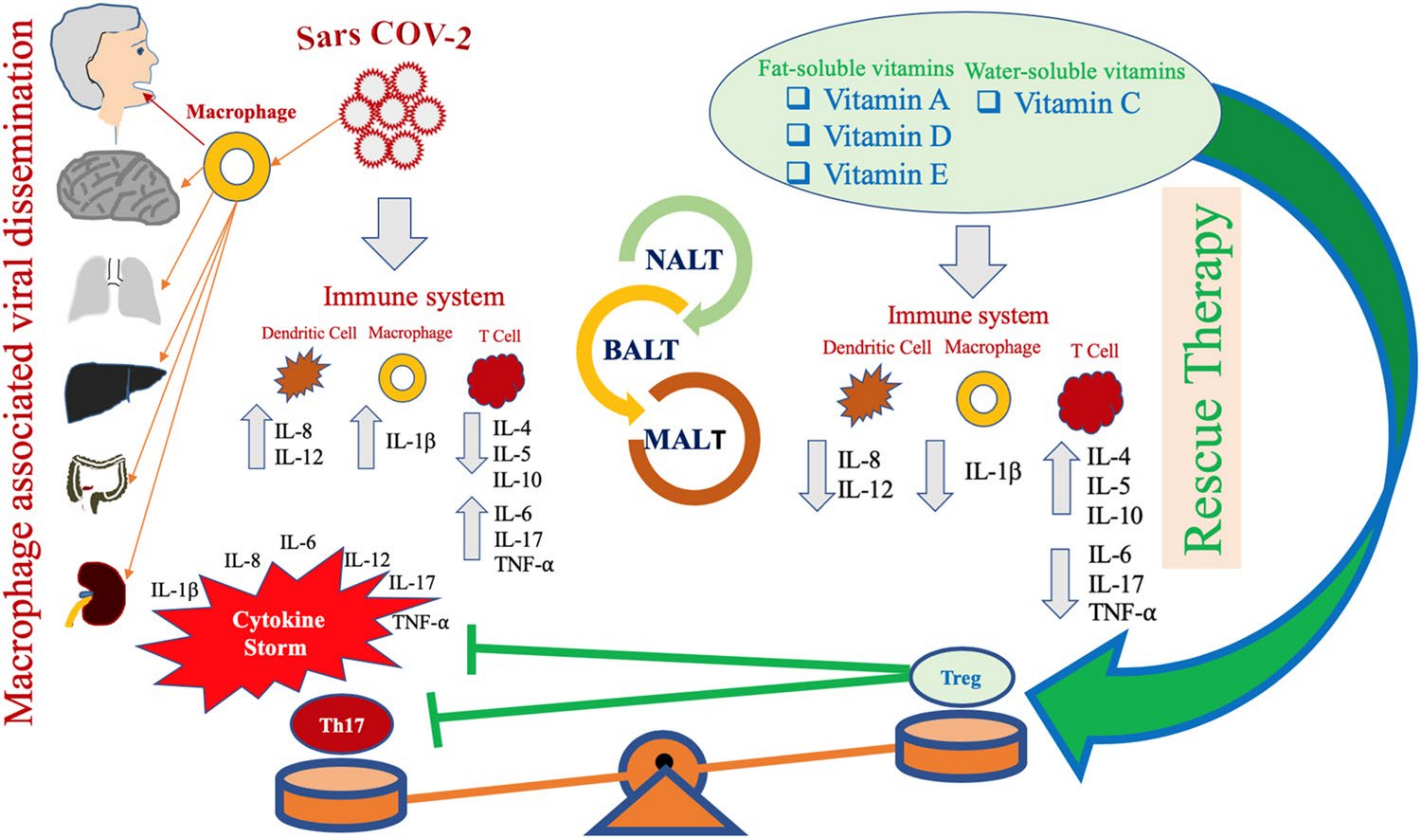
Possible pathogenesis in SARS-CoV-2 infection. The virus infects the human respiratory tract, entering the epithelial cells of the trachea, bronchi, bronchioles, lungs, as well as colonizing resident, infiltrating, and circulating cells of the immune system. Following this event, the virus spreads to cells of different organs, including the mucosa of the intestine, the epithelium of the renal distal tubules, and the neurons of the brain via infected circulating immune cells. The wide colonization of the various organs contributes to explaining the variety of symptoms. Based on this assumption, it may be hypothesized that infected circulating immune cells reach the nasal-associated lymphoid tissue (NALT), the mucosa-associated lymphoid tissue (MALT) and the bronchus-associated lymphoid tissue (BALT). Immune system function is severely impaired and a pneumonia with different degrees of severity may emerge in infected individuals, with possible fatal outcome. These subjects generally develop more severe clinical pictures and present a more elevated mortality in comparison with healthy subjects. Since several years ago, epidemiological studies have underlined that the elderly people as well as individuals with chronic diseases often present compromised immune functions even in normal situations and in times of non-medical emergency. These individuals have a lower serum concentration of fat-soluble and hydrosoluble vitamins. It is well known that these micronutrients play a crucial role for the normal functioning of the immune system. A, D, E and C vitamins, used not as simple supplements, but at pharmacological dosages may have a significant impact in counteracting the immune-suppressive activity of SARS-CoV-2 and may attenuate several aspects of the host’s excessive immune response against this pathogen. CoV-2 may promote a shift of the immune response towards a Th17 proinflammatory phenotype, causing the development of an unfavorable clinical evolution of the disease. Taking advantage from studies performed in patients with autoimmune diseases, such as rheumatoid arthritis and SLE, it is conceivable that the promotion of an anti-inflammatory response, mediated by the induction of a prevalent T-regulatory-cell phenotype, may represent a very promising strategy for the treatment of patients suffering from CoV-2-related infectious disease. Several therapeutic approaches have been proposed against these autoimmune diseases. These trials have shown that at pharmacological dosage, vitamins A, D, E and C may actively contribute to inhibit the proinflammatory immune response in these diseases, by inhibiting the Th17-mediated response and by promoting a T regulatory activity of the immune system
2. *The normal activity of the immune response both in its innate and adaptive arms*. An in-depth discussion about the regulatory role of vitamins A, D, E and C in the proper functioning of the immune system against pathogens is beyond the scope of this article. Therefore, we will present in brief our current understanding of the essential activities of each vitamin in modulating a broad range of immune processes and in regulating the immune response against pathogens. The coordinated cooperation of all these micronutrients is essential for the maintenance of an adequate homeostasis and of proper activity of the immune system.

## Vitamin D

1,25(OH)2VD3 acts on T cells down-regulating T-helper-1 (TH1)-cell cytokines, particularly IFNγ, and stimulating TH2-cell responses [99, 100]. This event is induced by the decrease of IFNγ release and by the enhancement of IL-4 production [101]. Furthermore, 1,25(OH)2 VD3 regulates effector T-cell differentiation by modulating antigen-presenting DCs, which decrease their synthesis of IL-12, a cytokine that promotes TH1-cell responses [91, 102]. 1,25(OH)2VD3 also exerts an important effect on the activity of the immune system, by counteracting TH17-cell responses and the differentiation of naive TH-0 cells into a TH-17 type, as this micronutrient is able, in part, to down-regulate several pro-inflammatory cytokines, including IL-6, IL-17 and IL-23 production [103, 104], and promotes the reciprocal differentiation and proliferation of forkhead box protein 3 (FOXP3) + regulatory T (TReg) cells [105, 106]. Furthermore, 1,25(OH)2VD3 decreases B-cell expansion, plasma-cell development and IgG secretion [107], probably by modulating the activities of antigen presenting-cells (APC) and by a means of a direct action on B cells [108]. In addition, 1,25(OH)2VD3 decreases the synthesis of IL-12 and simultaneously increases the production of IL-10 by DCs. Therefore, the TH1-cell response is shifted to a T regulatory type 1 (TR1) cell response. The results emerging from these experimental studies in patients with rheumatoid arthritis have provided the rationale for the use of 1,25(OH)2VD3 in association with other drugs [109], including the anti-IL-6 receptor monoclonal antibody (Tocilizumab), in the treatment of patients suffering from rheumatoid arthritis of various severity [110]. Interestingly, the best response (assessed by means of Diseases Activity Scores) to the therapy was obtained in patients with sufficient serum 25(OH)D levels (≥ 30 ng/mL) when tocilizumab was initiated, in comparison with patients with lower serum 25(OH)D levels (< 30 ng/mL). 1,25(OH)2VD3 primarily exhibits inhibitory activities on the adaptive immune response. However, in some circumstances, it may enhance the release of IL-1 by both monocytes and macrophages. Vitamin D is able to stimulate the intracellular type I IFN system, which exerts antiviral activities [111].

## Vitamin A (retinoic acid)

The term vitamin A indicates both retinol and its analogues, called retinoids, of which at least 1500 different types are known, both natural and synthetic in origin [112]. Carotenoids that contain at least one unsubstituted β-ionone ring (such as beta-carotene) are also considered to be precursors of vitamin A [113]. According to available data, vitamin A and its metabolites are able to regulate both innate and adaptive arms of the immune response, by increasing IL-2 secretion, and to modulate proliferation, differentiation and cytokine signaling as well as production [114]**,** all in T, B and antigen-presenting cells [112, 115]. Vitamin A metabolites also modulate more specific functional aspects of the immune response, such as the TH1–TH2-cell balance and the differentiation of TReg cells and TH17 cells. In particular, depending on the physical and biochemical composition of the microenvironment, vitamin A, in the presence of different concentrations of cytokines and transcription factors, stimulates TH 0 cells to assume a TH-2 phenotype [116], while inhibiting TH-1 subtypes. Furthermore, in the presence of an adequate concentration of transforming growth factor-β (TGFβ), retinoic acid promotes TReg-cell differentiation in peripheral tissues, whereas it inhibits the progression of lymphocytes from TH0 to pro-inflammatory TH-17 [117–119]. Retinoic acid displays gut imprinting ability on T and B cells and induces the differentiation of B cells to IgA-producing plasma-cells. A severe defect in intestinal immune responses [120] with enhanced mortality is detectable in patients with vitamin A deficiency. These subjects suffer from gastrointestinal and respiratory infections [121, 122]**.** On the other hand, vitamin A administration is associated with a significant reduction in diarrhea and mortality in children with HIV infection or malnourishment [123]. Moreover, vitamin A is able to stimulate the intracellular type I IFN system, which exerts antiviral activities [124].

## Vitamin E

Vitamin E is a strongly lipid-soluble compound with antioxidant properties and is detectable in elevated amounts in immune cells [125]. Scavenger activity is one of the most important actions of vitamin E, but this characteristic is not able to explain overall effects of this nutrient, as eight members in the vitamin E group (α-, β-, γ-, and δ-tocopherols and α-, β-, γ-, and δ-tocotrienols) exist in nature, with almost equal ability to quench free radicals. Each of these different forms of vitamin E sometimes acts distinctly from the other analogues, with not completely predictable activities [48]. Vitamin E may interact with several classes of enzymes, modulating their ability to bind to the plasma membrane and modifying a large series of cell signaling pathways. The families of enzymes modulated by vitamin E include protein kinases, protein phosphatases, lipid kinases, lipid phosphatases, lipid metabolic enzymes and enzymes involved in cAMP (cyclic adenosine monophosphate) metabolism. They regulate a wide spectrum of key cellular processes, including energy production, proliferation/apoptosis/death, protein synthesis, maintenance of quiescent status. Many of these enzymes are involved in crucial activities of the immune system and in inflammatory events, like cyclooxygenase-2 (COX-2), phospholipase A2 (PLA2), 5-, 12-, and 15-lipoxygenases (5-, 12-, 15-LOX), protein kinase C (PKC), protein kinase B (PKB/Akt), protein tyrosine kinases (PTKs). A wider discussion about all these aspects is beyond the aims of this paper and will not be carried on further, but the key role of vitamin E in the regulation of the inflammatory process has to be underlined [71]. Vitamin E and its metabolites have been shown to attenuate and limit inflammation by directly targeting COX-2 and 5-lipoxygenase [126]. This micronutrient displays additional functions as an effective regulator of the immune activity. Vitamin E supplementation above current dietary recommendations increases the activity of the immune system, confers protection against several pathogens and decreases the risk of infection, mainly in aged subjects [127]. Some cell-based, pre-clinical and clinical studies have evaluated the effects of vitamin E on immune system functions and on inflammation [128–130]. The most important effects of vitamin E supplementation on immune system activities include lymphocyte proliferation, post-mitogenic stimulation, an increase of delayed type hypersensitivity (DTH) response via stimulation of IL-2 production, a decrease of PGE2 via COX-2 inhibition, and a reduction of IL-6 release [131]. Furthermore, in both in vitro and in vivo studies vitamin E has been shown to improve natural-killer, naive T-lymphocytes, and dendritic cell activities, to promote initiation of T-cell activation signals as well as to rebalance IL-12 production and the Th1/Th2 ratio. Furthermore, vitamin E inhibits the production of pro-inflammatory cytokines, including IL-1, IL-6, TNF, and the chemokine IL-8, by monocytes and macrophages. Vitamin E is able to stimulate the intracellular type I IFN system, which exerts antiviral activities [57, 132–137].

## Vitamin C

Vitamin C also exerts a regulatory effect on immune system function; in particular, it has been reported to modulate the activity of T cells of the adaptive arm. Some studies have shown that vitamin C decreases mRNA expression of proinflammatory cytokines in vitro and inflammatory status in obese patients with hypertension in vivo. Dietary supplementation in these subjects reduced serum levels of high-sensitivity C-reactive protein, interleukin 6 (IL-6), fasting blood glucose (FBG) and triglyceride (TG) after 8 weeks of treatment [138]. In addition, the combined supplementation with vitamins C and E has been associated with the decrease of oxidative stress in patients with HIV infection, with a trend towards a reduction in viral load [74]. Furthermore, vitamin C supplementation, alone or in association with vitamin (trans retinoic acid) A, is able to generate stable antigen-specific regulatory T cells in animal models of autoimmune- or acute graft versus host-diseases [139, 140]. Favorable effects of vitamin C in alleviating the common cold as well as pneumonia have been reported in some controlled trials. Vitamin C is able to stimulate the intracellular type I IFN system, which exerts antiviral activities. Additional studies are needed to confirm the promising role of vitamin C in the treatment of patients with infections caused by bacteria, viruses, and protozoa [141].

## Conclusions

The lesson emerging from all these considerations, assumptions, and available data is that the different forms and metabolites of fat-soluble and hydrosoluble vitamins constitute a very complex mixture with multiple effects. Therefore, their activities should be not considered individually, but as a whole, taking into account that the final effect of each vitamin alone or in combination with the other micronutrients depends on the remodulation and on the rebalancing of the overall activities of the immune system and that these are not directly predictable. Therefore, a key general point emerges: fat-soluble and water-soluble vitamins possess pleiotropic regulatory effects on a large series of cell activities and represent powerful means of modulating and modifying various crucial cellular functions. On the basis of all these considerations it seems reasonable to hypothesize the use of vitamins A, D, E and C with preventive purpose, with the aim to restore immune system function in aged people at increased risk of infection-related mortality as well as for the treatment of patients suffering from SARS-CoV2-mediated acute infection [142]. Taking advantage from all these immunopathogenic assumptions as well as epidemiological and clinical observations, a possibly useful approach for the effective management of the health concern represented by SARS-CoV-2 should include the subdivision of the general people into two groups:

1. *Patients with acute infectious disease*, requiring an effective antiviral treatment. The therapeutic schedule for these patients may include: (a) antiviral therapy with current available drugs with reported effective antiviral effects in preliminary trials and studies. It may be hypothesized that this treatment should be administered as soon as possible to block viral replication as well as synthesis and release of viral proteins (mainly nucleocapsid and spike proteins) with the purpose of preventing the establishment of a self-maintaining and self-increasing robust pro-inflammatory loop, which leads to the “cytokine release syndrome” in the first phases of the disease; (b) immunomodulatory therapy, including (i) monoclonal antibodies against the IL-6 receptor (as proposed in very preliminary studies) and eventually against IL-1 and/or IL-8, as well as cyclo-oxygenase inhibitors, such as aspirin or FANS, with the purpose of blocking or preventing a strong inflammatory response and the release of additional cytokines and mediators of inflammation. This approach should also be started as early as possible. Unfortunately, no trials investigating the efficacy and safety of this treatment, as well as its optimal duration, are available; (ii) therapeutic schedules including the administration of liposoluble and hydrosoluble vitamins (such as A, D, E and C) on the basis of the well-known beneficial immunoregulatory and immunomodulatory roles of these micronutrients.
2. *General population without SARS-CoV-2-related acute infection*, including healthy or young individuals as well as aged-people or patients with chronic diseases. Epidemiological data show that elderly or persistently ill subjects are at a higher risk of death following SARS-CoV-2 infection, compared to younger or healthier individuals. Furthermore, available studies report that fat-soluble and water-soluble vitamins are necessary for correct functioning of the Immune System, and that in patients with different chronic infections, including HBV, HCV and HIV infections, vitamin A, D, E and C deficiency is associated with higher levels of viral replication and with higher titers of inflammatory cytokines, like IL-6 and TNF-α. Therefore, the supplementation of A, D, E and C vitamins among these classes of individuals may represent a possible preventive strategy with the aim of improving immune system function. The above-mentioned micronutrients possess multiple well-known nuclear and cytoplasmic targets in all the different types of mammalian cells, and they may modulate and regulate an elevated number of intra- and extracellular pathways. Therefore, these vitamins may contribute to modulating and restoring immune system functions and to preventing the cytokine release syndrome. Overall, these compounds may be considered not only as physiological substances but also as real drugs with potential useful or dangerous effects. Unfortunately, to date no studies have assessed the blood concentration of these liposoluble vitamins in patients with SARS-CoV-2; it is also unknown whether vitamin deficiency may be associated with a more severe course and outcome of this disease. Therefore, trials evaluating blood concentration of these compounds should be performed as soon as possible. Considering all these data and the possible side effects of these compounds, a dosage of blood liposoluble vitamins should be performed in patients with SARS-CoV 2 infection already in the early phases of the disease. Unfortunately, no studies have been designed to verify the real usefulness of this potential preventive strategy and to evaluate the possible effective dosages for each of these vitamins.

Many trials have been conducted with the aim to assess the usefulness of vitamins A, D, E and C for the treatment of patients with viral infectious diseases or of individuals with autoimmune diseases, such as rheumatoid arthritis and systemic lupus erythematosus (SLE) or of patients with infectious diseases. To the best of our knowledge, vitamins A, D, E and C, at the dosage used, have been demonstrated to possess safe profiles with no important side effects regarding potential therapeutic effect. Therefore, a possible use for these micronutrients might be considered in a multi-therapeutic treatment regimen.

Furthermore, it must be underlined that about 20% of patients with CoV-2 infectious disease develop interstitial pneumonia with severe tissue damage. It is unclear whether patients who recover from infection will have a complete recovery of lung injury with full restoration of tissue integrity or if persistent damage will develop, with possible evolution to a fibrotic tissue reaction and with the consequent emergence of a disability status in these patients. Vitamins D and A have been shown to be trophic for alveolar epithelial cells in in vitro studies, whereas vitamin C has not shown significant results in decreasing alveolar damage in a randomized, double-blind, placebo-controlled, multicenter trial compared to placebo in patients with sepsis and severe acute respiratory failure [143]. Overall, all these micronutrients may have a possible role in the mechanisms of alveolar tissue repair and their potential activities should be evaluated in future trials [36, 144].

In conclusion, in this article, we have provided a brief evaluation of the available data concerning this very life-threatening disease worldwide, known as SARS-CoV 2, and we have examined the crucial mechanisms potentially involved in the development of this severe illness. Based on our research we have identified the potential viral and host cellular targets and we have suggested a rationale for poly-therapeutic approaches. Further studies are strongly required to increase our knowledge of the immunopathogenesis of this disease, with the aim of contributing to the control of this public health emergency.

## Compliance with the standards

### Impact statement

(1) We certify that this work is novel. (2) What this research specifically adds to the literature: SARS-CoV-2 represents a public health emergency of international concern, but no effective therapy against this virus is currently available. Therefore, studies investigating the mechanisms involved in the pathogenesis of this infection and the possible strategy for its treatment are strongly requested. We have taken advantage from studies carried out in patients suffering from some autoimmune diseases, such as rheumatoid arthritis and systemic lupus erythematosus. Furthermore, it must be considered that proper functioning of the immune system requires the presence of adequate concentrations of several micronutrients like vitamins A, D, E and C. However, a large part of the population, mainly the elderly individuals, presents significant deficiencies in these micronutrients. For the first time, we have postulated the potential role of vitamins A, D, E and C in interfering with the inappropriate host’s inflammatory response caused by SARS-CoV-2 and in attenuating it. In particular, the pathogenesis of autoimmune diseases and their treatment may serve as a paradigm for a better understanding of the mechanisms involved in the development of the cytokine release syndrome (CRS). This life-threatening condition emerges in about 15–20% of patients with SARS-CoV2 and its control is necessary to prevent the fatal outcome in these subjects. In our paper, we have indicated the potential targets of vitamins A, D, E and C in modulating the intense immune response arising during SARS-CoV-2 infection. This effect is probably due to the direct down-regulation in the synthesis, release or function of cytokines (such as IL-6, IL-8, IL-17 and TNF-α) or enzymes (such as cyclooxygenase-2 and lipoxygenase) with strong pro-inflammatory activities as well as to the shift of Th17-mediated immune-response towards a T-cell regulatory phenotype, as observed in patients with autoimmune diseases. Taken together, these observations provide the rationale for the use of these micronutrients as part of a multi-treatment strategy against SARS-CoV-2 infection for the therapy of patients with acute forms of infection or as a strategy for improving immune system function. This approach could produce a beneficial effect on several groups of the population, mainly elderly people, and should be considered for these individuals with preventive purposes. Well-designed trials should be carried out with the aim to confirm or deny this strategy.
